# T10. HERITABILITY OF AMYGDALA ACTIVITY AND ITS GENOME WIDE ASSOCIATION WITH THE SCHIZOPHRENIA RISK LOCUS OF MIR137

**DOI:** 10.1093/schbul/sby016.286

**Published:** 2018-04-01

**Authors:** Tiziana Quarto, Giulio Pergola, Pasquale Di Carlo, Vittoria Paladini, Marco Papalino, Raffaella Romano, Antonio Rampino, Daniela Marvulli, Alessandro Bertolino, Giuseppe Blasi

**Affiliations:** University of Bari

## Abstract

**Background:**

It is well known that heritability plays a prominent role in risk for schizophrenia, and that this brain disorder is crucially characterized by emotional symptoms. Less known is how heritability shapes brain activity during emotion processing and whether this brain phenotype is also associated with genetic variation increasing risk for schizophrenia. Here, we implemented a multi-step, data-driven approach in order to assess the relevance of the link between heritability, genetic variation, and schizophrenia for brain activity during emotion processing.

**Methods:**

We investigated three samples of healthy individuals and one sample of schizophrenia (SCZ) patients: i) 28 healthy twin pairs (16 monozygotic and 12 dizygotic twin pairs); ii) 289 unrelated healthy participants (genome-wide association study - GWAS -discovery sample); iii) 90 unrelated healthy participants (replication sample); iv) 40 SCZ patients. During fMRI, participants approached or avoided threatening angry faces (explicit emotion processing). Intra-class correlations (ICC) between twin pairs and ACE models (A: additive genetics; C: common environment; E: unique environment) were used to identify regions of interest (ROIs) with heritable functional activity. Then, we extracted BOLD signal from these ROIs and conducted a GWAS on 565,137 single nucleotide polymorphisms (SNPs) (selected with the following criteria: minor allele frequency>0.15, Hardy–Weinberg equilibrium<0.001, linkage disequilibrium pruning r^2^>0.9) using robust linear models of allelic dosage corrected for multiple comparisons (Gao et al. 2008 Genetic Epidemiology). Finally, we assessed the effect of surviving SNPs in the replication sample of healthy individuals as well as in the sample of SCZ patients.

**Results:**

In healthy twins, we identified bilateral amygdala as the brain region with the highest heritability during explicit emotion processing as evaluated with our task (ICC=.79; h2=0.54; p<.001). The subsequent GWAS in healthy non-twins indicated that bilateral amygdala activity during the task was associated with a polymorphism close to miR-137 (rs1198575) (p=1.5 × 10–7), with the C allele corresponding to lower activity than the t allele. A similar effect was found in the replication sample (p=.01) and in patients with SCZ (p=.03).

**Discussion:**

Our data-driven approach revealed that amygdala activity as evaluated with our task is heritable. Furthermore, our results indicate that a polymorphism in miR-137 has genome wide association with amygdala response during emotion processing which is also replicated in two independent samples of healthy subjects and of patients with schizophrenia. Previous findings indicated that this polymorphism has genome-wide association with schizophrenia (Ripke et al. 2014). Other results reveal that miR-137 is a key regulatory neuronal factor linked to SCZ and involved in emotion processing (Cosgrove et al., 2017). Our findings are consistent with these previous findings and further highlight a crucial role for miR-137 in emotion processing and SCZ (Anticevic et al., 2012 Schizophr Bull).

